# A case of chronic neutrophilic leukemia and multiple myeloma showing the benefits of lenalidomide and cyclophosphamide therapy in treating both conditions

**DOI:** 10.1002/ajh.26670

**Published:** 2022-09-10

**Authors:** Kathryn McVinnie, Andrew Innes, Elisabet Nadal‐Melsio, Maria Atta, Simona Deplano

**Affiliations:** ^1^ Department of Haematology Hammersmith Hospital London UK; ^2^ Department of Molecular Pathology Hammersmith Hospital London UK

## 1 CASE PRESENTATION

**An 85‐year‐old woman was referred to hematology in 2019 for a persistent thrombocytosis who had been present for over 3 years. Her presenting blood parameters were hemoglobin 113 g/L, mean corpuscular volume 92.5 fL, white cell count (WBC) 12.6 × 10** ^**9**^ **/L, neutrophils 9.7 × 10** ^**9**^ **/L, monocytes 1 × 10** ^**9**^ **/L, and lymphocytes 1.7 × 10** ^**9**^ **/L. The patient reported symptoms from recurrent chest infections but no systemic upset, including no weight loss, fatigue, fevers, or night sweats. She had a past medical history of an unprovoked pulmonary embolus, breast cancer treated by wide local excision and radiotherapy, renal cell carcinoma managed by nephrectomy, and recurrent chest infections from bronchiectasis. Initial work up demonstrated iron deficiency with transferrin saturations of 6% and a ferritin of 62 μg/L. Her vitamin B12 was elevated at 1534 ng/L, with normal serum folate of 9.5 μg/L. Mutational analyses of 13 genes commonly mutated in myeloid malignancies, including Janus Kinase 2 (*JAK2)*, calreticulin (*CALR)*, thrombopoietin receptor (*MPL)*, and colony‐stimulating factor 3 receptor (*CSF3R)* was negative on peripheral blood testing. Oral iron was commenced.**

This initial presentation demonstrated appropriate work up for primary and secondary causes of thrombocytosis. The working diagnosis was reactive thrombocytosis secondary to iron deficiency and recurrent chest infections.

**Despite treatment of her iron deficiency, the thrombocytosis continued, so she underwent a bone marrow aspiration and trephine biopsy, which showed no evidence of a myeloproliferative neoplasm (MPN) but with some mild dysplastic changes mostly affecting the megakaryocytic lineage, which were thought to be age related. Subsequently, her platelet count normalized after 9 months of oral iron replacement (counts on December 6, 2019 hemoglobin 111 g/L, WBC 6.9 × 10** ^**9**^ **/L, neutrophils 5 × 10** ^**9**^ **/L, platelets 314 × 10** ^**9**^ **/L) and she was discharged from hematology care with advice for the general practitioner to continue long‐term low‐dose iron supplementation.**

The lack of concerning features on bone marrow examination was reassuring. The patient was treated with iron supplementation and resolution of the blood abnormalities was demonstrated. Usually at that stage causes of the prolonged iron deficiency would be investigated and gastroscopy and colonoscopy were offered prior to discharge from hematology, however, the patient declined these investigations.

**FIGURE 1:**
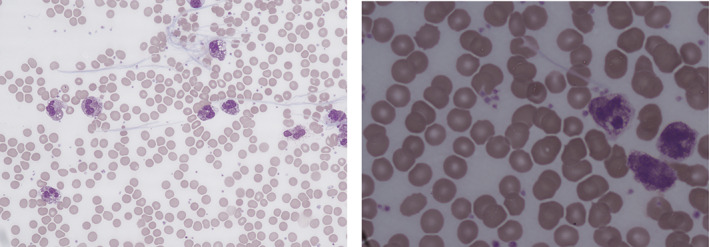
Blood films at ×100 and ×40 objective showing fibrin strands, thrombocytosis, neutrophil toxic granulation [Color figure can be viewed at wileyonlinelibrary.com]

**The patient was re‐referred in October 2021 following a progressive new neutrophilia and a recurrence of her thrombocytosis. She reported night sweats and lethargy, which were new in the previous few months. Her presenting blood counts were hemoglobin 109 g/L, WBC 39.7 × 10** ^**9**^ **/L, neutrophils 35.5 × 10** ^**9**^ **/L, monocytes 1.5 × 10** ^**9**^ **/L, and platelets 557 × 10** ^**9**^ **/L. She had an elevated erythrocyte sedimentation rate of 119 mm in 1 h and an increased ferritin of 315 μg/L. Renal function was stable (creatinine 92 μmol/L, urea 9.3 μmol/L, estimated glomerular filtration rate 49 ml/min/1.73 m** ^**2**^ **) and there was mild hypercalcaemia (2.66 mmol/L). Her blood film showed neutrophilia with predominantly mature neutrophils and band forms, with marked toxic granulation and vacuolation. Thrombocytosis, fibrin strands, and rouleaux formation were noted (Figure** **1** **)**.

With the above history and investigative findings, it was important to exclude recurrence of previous breast or renal cancer, a new malignancy or chronic inflammatory condition, as well as a working up for primary hematological causes. Whilst the patient agreed to undergo invasive investigations to reach a diagnosis, a balance of risk–benefit for potentially high risk procedures was necessary given this patient's age and her performance status of 2.

**FIGURE 2:**
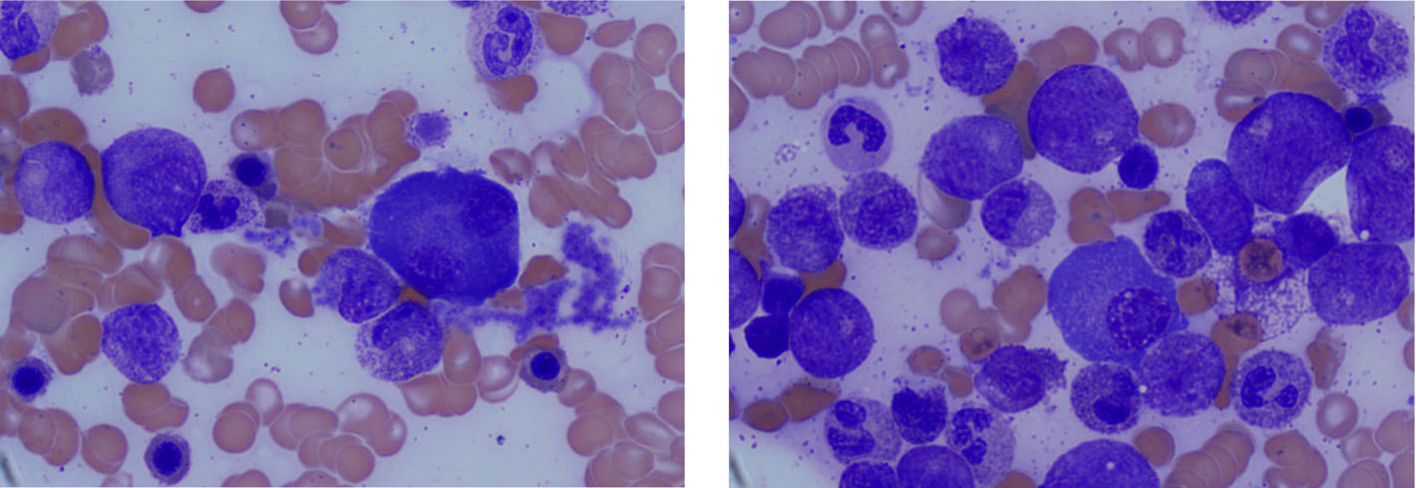
Bone marrow aspirate ×100 objective showing myeloid expansion and left shift of myeloid precursors with large plasma cells [Color figure can be viewed at wileyonlinelibrary.com]

**A computed tomography (CT) of the neck, chest, abdomen, and pelvis was performed, which showed new lucent bony lesions in the T4 vertebral body and posterior left 4th rib but no evidence of primary malignancy. Tumor markers demonstrated slight increases in carcinoembryonic antigen (6 μg/L), cancer antigen (CA) 15–3 (45 μg/L) and CA 125 (39 kunits/L) with normal CA19‐9 (<2 kunits/L). Protein electrophoresis demonstrated an immunoglobulin G (IgG) lambda paraprotein of 12 g/L. Lambda light chains were increased at 148.6 mg/L with kappa light chains of 18.5 mg/L, kappa: lambda ratio 0.12. A positron emission tomography/CT was performed, which demonstrated the lytic deposits at T4 and left posterior rib, with appearances suspicious of focal diffuse myelomatous marrow disease. A repeat bone marrow aspirate demonstrated a hypercellular marrow with myeloid expansion, left‐shifted myelopoiesis although no excess of blasts, and 7%–10% plasma cells, which were lambda restricted. The plasma cells were large and atypical with prominent nucleoli. Cytogenetic analysis showed a normal karyotype. In molecular studies, two somatic *CSF3R* mutations were discovered (in both peripheral blood and bone marrow)—a missense T618I mutation and a truncating S810fs mutation with variant allele frequencies (VAF) of 42% and 43%, respectively. There were no mutations in *JAK2* (exons 12 and 14), *CALR*, or MPL and fluorescence in situ hybridization (FISH) showed no *BCR‐ABL1*, *FIPLI‐PDGFRA*, *PDGFRB*, or *FGFR1* rearrangement (Figure** **2** **).**

Following the above investigations, two diagnoses were made: the patient fulfilled the criteria for both IgG lambda multiple myeloma and chronic neutrophilic leukemia (CNL). Although the plasma cell percentage in the bone marrow was low, this was in the context of markedly expanded myelopoiesis. Although a leukemoid neutrophilic reaction in the context of multiple myeloma is also possible, the presence of the *CSF3R* mutations does not support this. Pursuing biopsy of the lytic lesions in this case would not have changed management, and the mildly elevated tumor markers were not felt to be significant. Given how symptomatic the patient was, a discussion occurred about treatment. Treatment of the multiple myeloma was felt to be most appropriate in the first instance, including cytotoxic chemotherapy, which could also benefit the CNL.

**Attenuated lenalidomide (10 mg daily for 21 days), dexamethasone (10 mg weekly), and cyclophosphamide (250 mg weekly) were commenced (rituximab, cyclophosphamide, and dexamethasone [RCD] regimen). Within one cycle, this led to resolution of the neutrophilia and thrombocytosis (to 2.9 × 10** ^**9**^ **/L and 242 × 10** ^**9**^ **/L respectively). The chemotherapy was well tolerated by the patient, other than worsening of her anemia with hemoglobin falling to 90 g/L, requiring a blood transfusion for symptomatic relief. After two cycles of oral cyclophosphamide, this was discontinued due to the normalization of her full blood count. Since then, she has continued on lenalidomide and dexamethasone only, and has now been followed up for over eight cycles of chemotherapy in total.**

**FIGURE 3:**
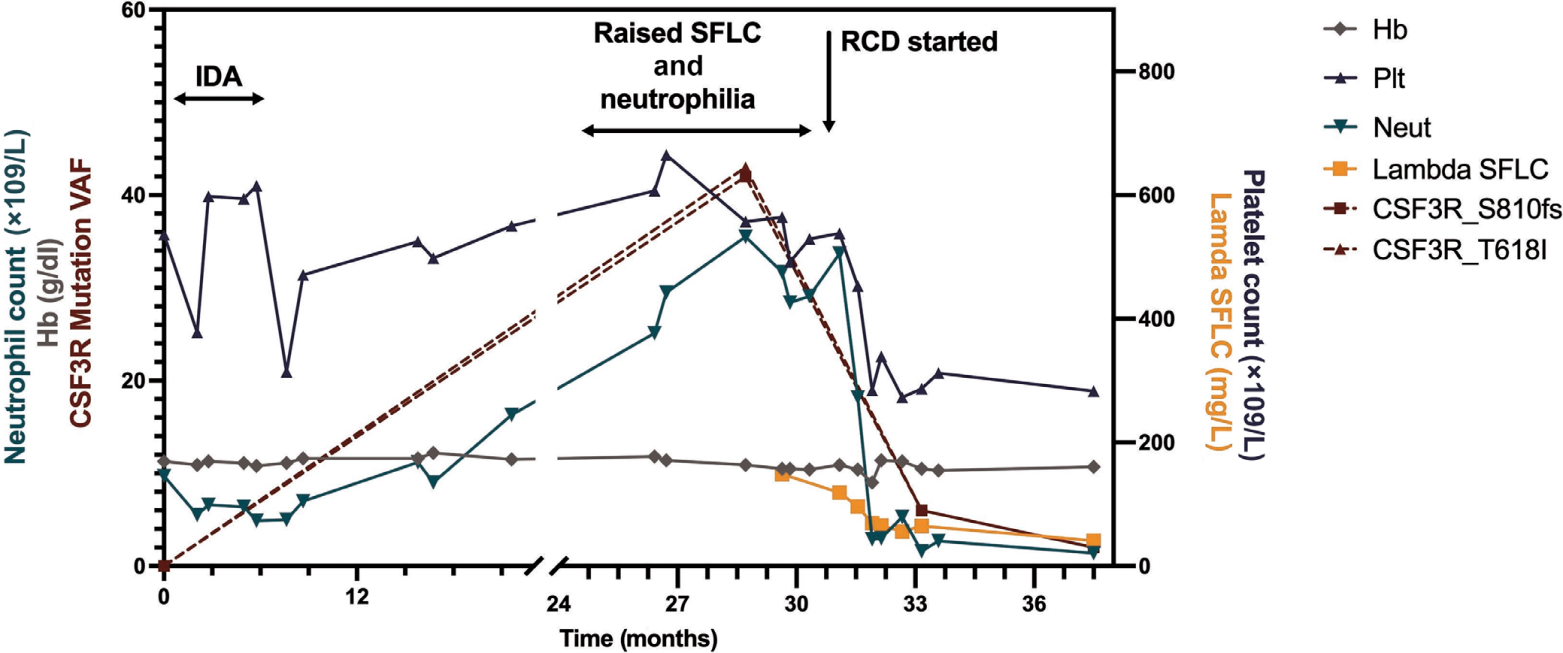
Graph showing changes over time in neutrophil count, hemoglobin, CSF3R VAF and platelet count from original presentation, to chronic neutrophilic leukemia and multiple myeloma diagnoses, and after 3 and 6 cycles of chemotherapy. CSF3R_S810fs, colony stimulating factor three receptor frameshift mutation; CSF3R_T618I, colony stimulating factor receptor missense mutation; Hb, hemoglobin (g/dL); IDA, iron deficiency anemia; Neut, neutrophil count (× 10^9^/L); Plt, platelet count (× 10^9^/L); RCD, lenalidomide, cyclophosphamide, dexamethasone chemotherapy; SFLC, serum free light chains (mg/L); VAF, variant allele frequency; Wt, wild type [Color figure can be viewed at wileyonlinelibrary.com]

**Repeat molecular studies after three chemotherapy cycles demonstrated a marked reduction in the VAF of the two *CSF3R* mutations detected initially, which both fell to 6% (from 42% and 43%). A further reduction to 2% was observed after completion of 6 cycles of chemotherapy. Her IgG lambda paraprotein remained low and stable at 3 g/L with lambda light chains of 41 mg/L and a normal kappa: lambda ratio. The figure below shows how some of the patient's blood parameters have changed over time, with marked improvement after chemotherapy and a sustained response, which has continued after cessation of the cyclophosphamide. The patient is also symptomatically much improved (Figure** **3** **).**

## 2 DISCUSSION

CNL is a MPN, which is diagnosed when the WBC count is above 25 × 10^9^/L, of which greater than 80% are neutrophils with less than 10% neutrophil precursors.^1^ There must also be no dysplasia or other identifiable MPN.^1^ Mutations in the *CSF3R* gene are associated with its pathogenesis.^2^ Treatment options for CNL include ruxolitinib, hydroxycarbamide, and dasatinib, however, there are no randomized controlled trials for CNL demonstrating their efficacy.^3^ CNL is frequently an aggressive disease, which can progress to acute leukemia (mean time to progression in one case series of 21 months) with a median survival of 21 months.^4^ Acute transformation is usually treated with more traditional acute leukemia regimens, depending on patient fitness, although outcomes remain poor.

The *CSF3R* missense mutation changing threonine to isoleucine at position 618 (T618I) is the commonest mutation found in CNL and causes ligand‐independent activation of the granulocyte colony‐stimulating factor receptor.^2^ Truncating mutations in the cytoplasmic tail, such as the frameshift mutation at position 810 (S810fs) present in this case, result in constitutive overexpression of the receptor and ligand hypersensitivity.^5^ The membrane proximal missense mutations (e.g., T618I) are commonly seen alone in CNL, although co‐existing truncating mutations are seen in up to 25% of cases.^2^ In contrast to T618L mutations, truncating mutations are not leukemia initiating in murine models but accelerate leukemia development in the presence of *PML‐RARA* translocation, suggesting that they may be secondary events.^6^ In addition, the truncating mutations are also frequently seen at the point of transformation to acute myeloid leukemia (AML) in a sub‐group of patients with congenital neutropenia treated with granulocyte colony‐stimulating factor.^7^ Whether it has the same effect in CNL should be the focus of future research. In addition, it has been suggested that the truncating mutation may be responsible for the neutrophil toxic granulation, which is not always present in CNL.^8^

There are some case reports of an association between plasma cell neoplasms and neutrophilia, however many of the cases in the literature were reported prior to the identification of the *CSF3R* mutation as a pathognomonic feature of CNL, and it has now been suggested that many of these cases represent plasma cell‐driven neutrophilia, possibly by the production of granulocyte colony‐stimulation factor and other cytokines by the plasma cells.^8^, ^9^, ^10^ However, in this case, the presence of the *CSF3R* mutation indicates there are two distinct diagnoses in play, namely CNL and a plasma cell neoplasm, rather than merely a reactive neutrophilia driven by abnormal plasma cells, and this is a more unusual finding.

Lenalidomide is an immunomodulatory drug (IMiD), which is used to treat both multiple myeloma and myelodysplastic syndromes (MDS). In myeloid disorders, it was first shown to be beneficial in patients with MDS with chromosome 5q31 deletion.^11^ The mechanism of action of IMiDs in hematological disease is not completely understood and may be via more than one mechanism, including immunomodulation and anti‐inflammation. In addition, IMiDs have been shown to bind to cereblon (CRBN), which is the substrate receptor of the CRL4 E3 ubiquitin ligase receptor.^12^ When bound to an IMiD, CRBN changes the targets for this ubiquitin ligase complex, causing ubiquitination of some target proteins, including IKZF1 and IKZF3 (also called Ikaros and Aiolos, respectively), which leads to their downregulation.^13^, ^14^ IKZF1 and IKZF3 are transcription factors, which play a role in the pathogenesis of multiple myeloma but are also important in myeloid disorders; recently, Fang et al. demonstrated that lenalidomide's mechanism of action in AML and MDS is to induce cell cytotoxicity through CRBN and IKZF1 via a calcium‐dependent pathway.^14^, ^15^ Studies have shown the possible role of lenalidomide in other myeloid disorders, such as AML, but to date there are no studies suggesting a mechanism of action for patients with both CNL and plasma cell dyscrasia that the authors are aware of.^16^, ^17^

An important learning point from this case is remembering to repeat investigations again on subsequent presentations even if they were unremarkable the first‐time round, or previous diagnoses have been made. For example, this patient was negative for *CSF3R* mutation on her first presentation but had mutations detected when repeated on her second presentation.

Given the frequency of plasma cell neoplasia in the elderly population it is not unreasonable to suggest this dual finding is coincidental, however further research is required to assess whether there is a closer association between these two diseases, as this may also help us find new treatment targets, particularly in CNL, which continues to be difficult to manage in many patients. The improvement in this patient's neutrophilia and *CSF3R* mutation VAF with RCD chemotherapy are promising signs, and further research is required to understand whether this regimen could play a role as a future treatment in patients with both CNL and plasma cell dyscrasia.

## CONFLICT OF INTEREST

The authors have no conflicts of interest to declare.

## Data Availability

Data sharing not applicable—no new data generated.
